# CD4 ^+^ T Cells Mediate Dendritic Cell Licensing to Promote Multi‐Antigen Anti‐Leukemic Immune Response

**DOI:** 10.1002/cam4.70508

**Published:** 2024-12-27

**Authors:** Luis Gil‐de‐Gómez, Joseph J. Mattei, Jessica H. Lee, Stephan A. Grupp, Gregor S. D. Reid, Alix E. Seif

**Affiliations:** ^1^ Division of Oncology The Children's Hospitial of Philadelphia Philadelphia Pennsylvania USA; ^2^ Department of Molecular Biology University of Cantabria School of Medicine Santander Cantabria Spain; ^3^ Department of Pediatrics The Perelman School of Medicine, University of Pennsylvania Philadelphia Pennsylvania USA; ^4^ Michael Cuccione Childhood Cancer Research Program BC Children's Hospital Research Institute Vancouver British Columbia Canada; ^5^ Department of Pediatrics University of British Columbia Vancouver British Columbia Canada

**Keywords:** acute lymphoblastic leukemia, CD4^+^ T cells, dendritic cells, Neoantigens

## Abstract

**Background:**

Single antigen (Ag)‐targeted immunotherapies for acute lymphoblastic leukemia (ALL) are highly effective; however, up to 50% of patients relapse after these treatments. Most of these relapses lack target Ag expression, suggesting targeting multiple Ags would be advantageous.

**Materials & Methods:**

The multi‐Ag immune responses to ALL induced by transducing cell lines with xenoAgs green fluorescent protein and firefly luciferase was elucidated using flow cytometry, ELISA, and ELISpot assays.

**Results:**

In our model, leukemia responsiveness correlates with in vivo CD4+ T cell activation and DC maturation, supporting a role for DC licensing. In contrast, tolerance is characterized by in vivo increased expression of negative immune checkpoints (IC) which may suppress rather than license DC. In vitro assays confirm the ability of CD4+ T cells from leukemia‐responsive mice to promote robust maturation of naïve bone marrow DC in the presence of non‐immunogenic leukemia antigens.

**Conclusion:**

Together these findings support a CD4+ T cell‐mediated mechanism of DC licensing to promote multi‐Ag immune responses that may augment current targeted immunotherapies and avoid relapses in treated children with ALL.

## Abstract 2

CD4^+^ T cell‐mediated mechanism of DC licensing can promote multi‐Ag immune responses that may augment current targeted immunotherapies.

## 1 Introduction

Single antigen (Ag)‐targeted immunotherapies for acute lymphoblastic leukemia (ALL) can overcome some of the natural barriers to generating anti‐ALL immunity, but up to 50% of patients relapse within a year of treatment [1, 2]. Most relapses after these treatments are target Ag‐negative, highlighting the need to identify ways to induce broader immune responses that target additional leukemia Ags. However, most children who develop ALL have abnormal cells bearing leukemia‐initiating mutations at birth, [3, 4, 5] thus immunologic tolerance to early‐occuring leukemia Ags is likely to be a hurdle to generating broad/multi‐antigen anti‐ALL immunity in children. Using the Eμ‐ret mouse model of ALL, which generates pre‐leukemic B cell precursors in utero, [6, 7] we previously demonstrated the impact of early‐life leukemia‐associated antigen expression on the generation of durable immune protection from ALL. [8] In wild type (wt) BALB/c mice, we observed apparent epitope spreading, with recognition of non‐immunogenic ALL Ags contributing to durable protection. In contrast, while Eμ‐ret transgenic BALB/c mice mounted a primary response to GFP/luc‐ALL similar to wt BALB/c, they succumbed to leukemia progression over subsequent weeks. These results revealed that Eμ‐ret transgenic mice, in which early leukemia‐driver genes are expressed in utero, were unable to achieve effective epitope spreading.

As a contribution from diversified immune responses to the maintenance of remission would have clear translational relevance for childhood ALL, we now investigate the settings in which non‐immunogenic leukemia‐associated Ags are recognized and the impact of prenatal leukemia‐initiating events on this response. To do this, we again made use of our xenogeneic antigen (xenoAg) model in which green fluorescent protein and firefly luciferase (GFP/luc‐ALL) serve both as bioluminescent/fluorescent reporters and as strongly immunogenic Ags: expression of GFP/luc in Eμ‐ret ALL blasts results in T cell‐mediated leukemia control after transplant into syngeneic BALB/c mice. Using a model that relies on in situ antigen presentation cells (APC) to trigger anti‐ALL responses, rather than a re‐directed T cell approach (e.g., CAR‐T), enabled comparison of the variables that affect APC and T cell pathways for both strong and non‐immunogenic antigens.

## 2 Material and Methods

### 2.1 Cell Lines

Cell line 289, derived from a spontaneous Eμ‐ret mouse leukemia, [6, 7] was transduced with a self‐inactivating lentiviral construct encoding green fluorescent protein and firefly luciferase (GFP/luc) to generate stable GFP/luc‐expressing variants [9, 10, 11]. All cell lines were routinely tested periodically for *Mycoplasma*. Primary ALL blasts were obtained from leukemias arising spontaneously in Eμ‐ret mice in our colonies at the Children's Hospital of Philadelphia (UBC) and the University of British Columbia (UBC).

### 2.2 Mouse Strains

C.FVB‐Tg(CAG‐luc,‐GFP)L2G85Chco/Fath transgenic (GFP/luc‐transgenic) and wild type (wt) BALB/c mice (RRID:IMSR_ORNL:BALB/cRl) were obtained from Jackson Laboratory (Bar Harbor, ME). Eμ‐ret‐transgenic, GFP/luc‐transgenic, and Eμ‐ret/GFP/luc double transgenic BALB/c colonies were maintained at CHOP and UBC under specific pathogen‐free conditions and were ≥ 5 weeks old for experiments. For experiments using colony mice, both males and females were randomized to treatment arms with a goal of maintaining a similar age and sex distribution among arms. Investigators were not blinded to mouse genotype. Bioluminescence imaging to follow leukemia development was performed using a Xenogen Spectrum or a Spectral Instruments Imaging Ami X camera system and analyzed using respective manufacturers' software [12].

### 2.3 In Vivo Experiments

For in vivo experiments, we adoptively transferred 10^6^ ALL cells by tail vein injection and mice were sacrificed at day 20. Splenocytes were isolated and stained. FACS analysis was performed using different panels. For APC characterization anti–B220‐APC‐Cy7, anti‐CD11c‐APC, anti‐CD11b‐PerCP, anti‐CD80‐PE, anti‐CD40‐PE‐Cy7, anti‐F4/80‐BV510, anti‐MHCII‐APC‐Cy7, anti‐PD‐L1‐PercP‐Cy5.5, anti‐PDCA‐1‐PE, anti‐Ly‐6C‐PerCP (BioLegend, San Diego, CA) were used: for T cell characterization anti‐CD3‐APC, anti‐CD4‐BV510, anti‐CD8‐APC‐Cy7, anti‐PD‐1‐PerCP‐Cy5.5, anti‐CTLA‐4‐PE (BioLegend, San Diego, CA), FACS analysis was performed with CFlow Plus Analysis software (BD Biosciences, San Jose, CA).

### 2.4 BMDC Generation

For bone marrow dendritic cells (BMDC) generation, bone marrow cells were harvested from the naïve BALB/c mouse femur and the resulting cells were seeded in 6‐well plates and incubated with granulocyte‐macrophage colony‐stimulating factor (GM‐CSF) and IL‐4 at concentrations of 10 ng/mL for 7 days. Media containing each treatment were replaced every 2 days. On day 7, cells were collected and adjusted to 1 × 10^6^ cells/mL.

### 2.5 In Vitro Experiments

For in vitro experiments, total T cells and CD4^+^ T cells were collected by using CD3^+^ and CD4^+^ T cell isolation kits (StemCell, Vancouver, Canada), respectively. Isolated cells were cultured with BMDC and GFP/luc‐modified and unmodified ALL cells and incubated with 10 μg/mL of CpG oligodeoxynucleotides 1826 (CpG; Invivogen, San Diego, CA) for 24 h at 37°C. Previously, target cells (GFP/luc‐modified and unmodified ALL cells) were treated with mitomycin C (10 mg/mL) and incubated at 37°C for 2 h. IL‐12 p70 was measured with Quantikine ELISA kit (R&D Systems, Minneapolis, MN). IFN‐γ was measured by ELISpot assay. 10^5^ T cells were plated in 96 well multiscreen immobilon‐P (MAIP) filtration plates (Millipore Sigma, St. Louis, MO) previously coated overnight with a 2.5 μg/mL solution of rat anti‐mouse IFN‐γ (BD Biosciences, Franklin Lanes, NJ) and incubated overnight at 37°C. The assay was carried out using R10 medium containing RPMI with HEPES and glutamine, 10% FBS, 100 U/mL penicillin, 100 mg/mL streptomycin, and 55 μM 2‐mercaptoethanol (Gibco, Thermo Fisher, Waltham, MA). The plates were washed 3 times with sterilized PBS and blocked with R10 for 2 h before cells were plated. Spots were detected following the instructions from ELISpot kit.

### 2.6 Inclusion Criteria and Data Analysis

Inclusion criteria were based on strain and age. Eμ‐ret‐transgenic mice were excluded if older than 6 weeks. Sample sizes were selected based on results of pilot studies, and arms were occasionally unequal due to availability of transgenic mice bred in‐house. All animal experiments were performed in accordance to Institutional Animal Care and Use Committee (IACUC)‐approved protocols at CHOP and UBC. All analyses are two‐sided, where applicable. Statistical analyses were performed using GraphPad Prism for Mac, version 9 (La Jolla, CA).

## 3 Results and Discussion

**FIGURE 1:**
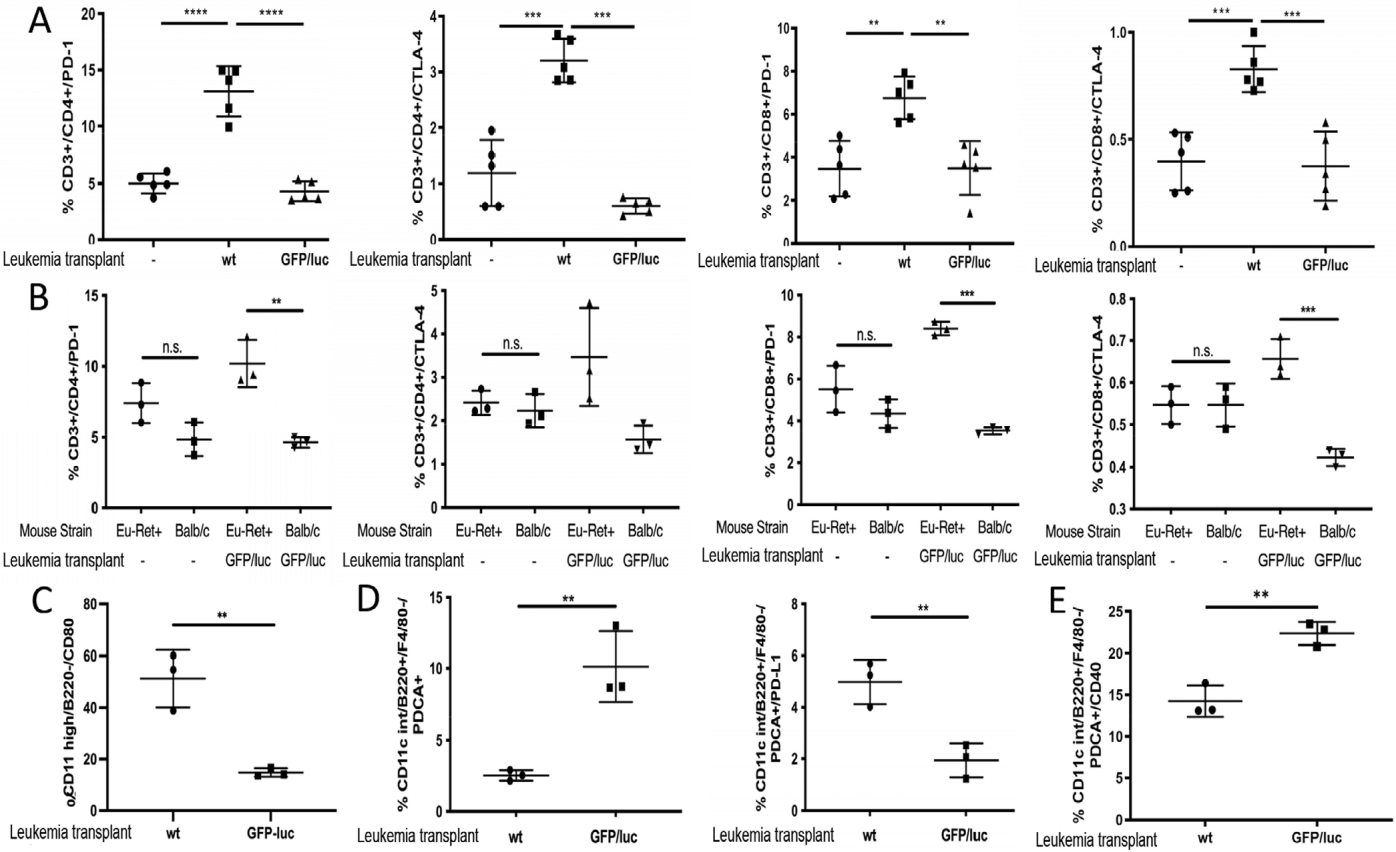
T cell response to transplanted leukemia is decreased in the setting of IC expression. (A) ICs PD‐1 and CTLA‐4 are expressed at higher levels on CD3^+^/CD4^+^ and CD3^+^/C8^+^ T cells harvested at day 20 from spleens from non‐responsive mice. One‐way ANOVA. (B) Early‐life exposure to the RET fusion protein (rfp) transgene results in increased expression of negative checkpoint inhibitors CTLA‐4 (on CD3^+^/CD4^+^ T cells) and PD‐1 (on CD3^+^/CD4^+^ and CD3^+^/C8^+^ T cells). Fresh splenocytes from transgenic (Eμ‐ret+) and wt (Eμ‐ret−) mice were stained before and 20 days after they were injected with GFP/luc leukemia cells. 2‐way ANOVA. (C) Upregulation of negative checkpoint regulators on CD3^+^ cells from non‐responsive mice correlates with upregulation of its ligand CD80 on conventional DC (cDC). Unpaired *t*‐test. (D) By day 20, plasmacytoid DC (pDC) are less abundant in the spleen from wt non‐responsive mice (left panel). Lack of responsiveness is also supported by the significant overexpression of PD‐L1 on pDCs from wt non‐responsive mice (right panel). Unpaired *t*‐test. (E) The activation of pDC is supported by the significant overexpression of CD40 after 14 days of exposure to GFP/luc ALL cells. *p < 0.05; **p < 0.01, ***p < 0.001.

We first evaluated the role of negative checkpoint regulators, such as programmed cell death protein‐1 (PD‐1) and cytotoxic T‐lymphocyte antigen‐4 (CTLA‐4). These IC have been implicated in ALL escape from targeted therapy [13, 14, 15]. However, PD1‐blockade alone is insufficient to restore full immune surveillance for ALL. [15]. Thus, we hypothesized that checkpoint regulators were limiting T cell response to non‐immunogenic ALL Ags. Both CTLA‐4 and PD‐1 were upregulated on CD3^+^/CD4^+^ and CD3^+^/C8^+^ splenic T cells harvested at day 20 from mice exposed to wt ALL (non‐responsive), but not on naïve T cells or T cells from GFP/luc‐ALL‐exposed BALB/c mice (responsive) (Figure 1A). Notably, T cell expression of both CTLA‐4 and PD‐1 were increased in Eμ‐ret mice receiving GFP‐luc leukemia cells, in contrast to naïve BALB/c (Figure 1B), further implicating these IC in the failure to respond to non‐immunogenic leukemia Ag. Although the functional status of these cells remains to be determined, the elevated IC expression on T cells from mice with progressing ALL is consistent with reports of higher PD‐1 and CTLA‐4 on T cells from ALL patients at diagnosis [16, 17]. While the observed changes in expression were modest, especially in the case of CTLA‐4, our previous observation of improved survival of wt‐ALL‐challenged Eμ‐ret transgenic mice after short‐term treatment with CTLA‐4 blockade suggests they have functional implications [8].

Based on these results, we evaluated the ligands of CTLA‐4 and PD‐1 present on APC surfaces. In contrast to CD80, which shows higher upregulation of expression on splenic conventional dendritic cells (cDC) from non‐responsive mice (Figure 1C), no difference in PD‐L1 expression on cDCs from leukemia‐responsive and non‐responsive mice was observed (Figure S1). We next investigated the effect of exposure of ALL‐presented xenoAgs on plasmacytoid DCs (pDC) (CD11c^low^, F4/80^−^ PDCA‐1^hi^, Ly‐6C^hi^) (Figure S2A). This subpopulation efficiently promotes cross‐presentation by recruiting and activating cDCs [18]. On day 20 after GFP/luc‐ALL exposure, wt BALB/c mice showed increased frequencies of splenic pDCs compared to mice who received wt ALL (Figure 1D left). However, in contrast to cDCs, the expression of PD‐L1 is lower on pDCs from responsive mice, suggesting exposure to immunogenic xenoAgs results in decreased negative checkpoint regulatory activity (Figure 1D right). To further evaluate pDC stimulation, we measured the expression of the engaging activator CD40. By day 14 after tumor challenge, CD40 expression is already upregulated on responsive mice (Figure 1E). These results suggest the activation of both cDCs and pDCs contribute to effective anti‐ALL responses, and increased IC expression is associated with non‐reposniveness.

**FIGURE 2:**
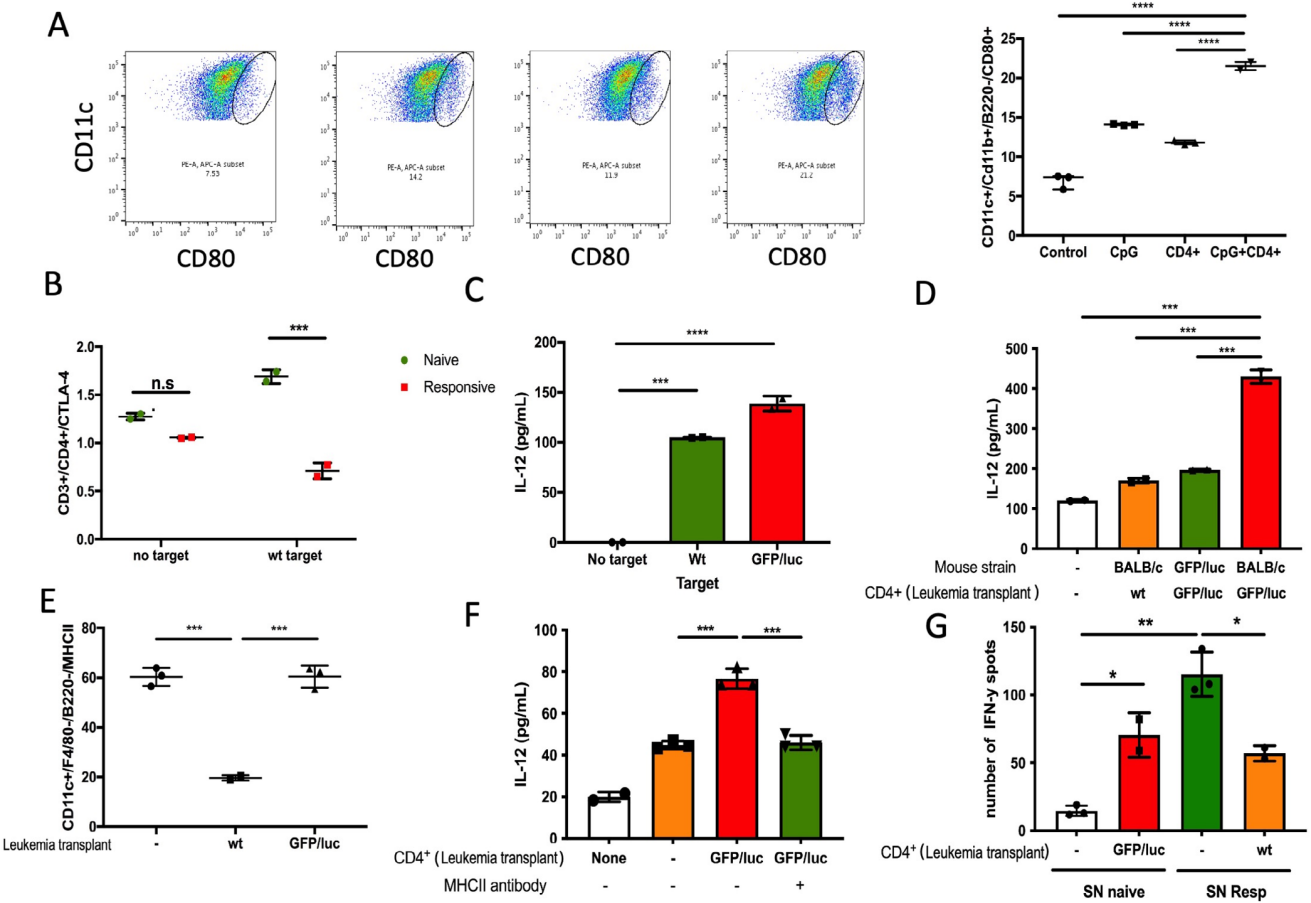
CD4^+^ T cells license naïve BMDC in presence of non‐immunogenic leukemia Ags but does not overcome tolerance. (A) CD4^+^ T cells from responsive mice activate naïve BMDC in presence of wt target cells to the same degree as CpG, as measured by surface CD80 upregulation. When CD4^+^ T cells and CpG are combined, there is an additive effect. One‐way ANOVA (B) CTLA‐4 is downregulated on CD3^+^/CD4^+^ T cells from GFP/luc‐ALL‐responsive mice after exposure to non‐inmunogenic ALL Ags. 2‐way ANOVA. (C) APC licensing is Ag specific. Absence of leukemia target cells results in no IL‐12 production from BMDC co‐cultured with CD4^+^ T cells from responsive mice and CpG. One‐way ANOVA. (D) CD4^+^ T cells from tolerant and non‐responsive mice are not able to promote APC maturation as responsive mice do in presence of CpG and wt ALL target cells. 2‐way ANOVA. (E) cDC from BALB/c non‐responders to wt ALL show downregulated MHC II in vivo. One‐way ANOVA. (F) BMDC licensing measured by IL‐12 production is abrogated by using MHC II blocking antibody. Absence of CD4^+^ T cells (white bar) and naïve CD4^+^ T cells (orange bar) were used as controls. Every condition includes incubation with wt target cells and CpG for 24 h. 2‐way ANOVA (G) High IL‐12‐containing supernatants collected from licensed BMDC (SN Resp) are able to promote the activation of naïve T cells (green bar) but tolerance abrogates this effect measured by IFN‐γ release (orange bar). 2‐way ANOVA: *p < 0.05; **p < 0.01, ***p < 0.001, ****p < 0.0001.

We have previously reported that recognition of non‐immunogenic leukemia Ags is achieved in the setting of GFP/luc‐directed or CpG ODN‐induced T cell responses against Eμ‐ret ALL [8, 19]. When combined with the increased checkpoint regulator expression shown in Figure 1, these findings led us to hypothesize that the lack of response to non‐immunogenic leukemia Ags in wt ALL‐challenged Eμ‐ret mice is a consequence of leukemia Ag‐tolerant CD4^+^ T cells failing to license APC to cross‐prime CD8^+^ T cells [20, 21]. Although ligation of CTLA‐4 induces T cell anergy, co‐stimulatory interactions between CD80 and CD28 at early time points are required for proliferative reponses. Therefore, we measured CD80 expression on bone marrow dendritic cells (BMDC) from naïve mice cultured for 7 days. Naïve BMDC were stimulated with the TLR9 agonist CpG oligonucleotides (ODN), CD4^+^ T cells from responsive mice, or both, in the presence of wt leukemia target cells for 24 h. CD80 was upregulated in the presence of either CpG or leukemia‐responsive CD4^+^ T cells (Figure 2A), and the combination of CpG and responsive CD4+ T cells resulted in even higher CD80 on BMDCs. To evaluate the role of CTLA‐4 as a CD80 ligand in the protective immune response after xenoAg exposure, splenocytes from naïve and GFP/luc‐ALL challenged BALB/c mice were exposed to wt ALL target cells for 24 h. In contrast to naïve CD3^+^ T cells, CD4^+^ cells from responsive mice show downregulation of CTLA‐4 (Figure 2B). To confirm APC licensing, we tested IL‐12 levels in BMDC culture supernatants. First, we tested the Ag‐dependent activation by CD4^+^ T cells. We co‐cultured CD4^+^ T cells from responsive mice with naïve BMDC stimulated with CpG in absence of Ags or in the presence of wt or GFP/luc‐ALL target cells and measured IL‐12 production. CD4^+^ T cells from responsive mice were able to activate naïve BMDC, resulting in robust IL‐12 production in the presence of both ALL target cells, but this response is not observed in absence of Ags (Figure 2C). Further, CD4^+^ T cells from responsive mice increased IL‐12 production by BMDC in the presence of wt ALL targets and CpG, but CD4^+^ T cells from naïve or non‐responsive mice did not (Figure 2D). The same pattern of IL‐12 production was observed in the absence of CpG (Figure S1). CD4^+^ T cells from GFP/luc‐transgenic (xenoAg‐tolerant) mice also failed to activate APCs. This indicates that Ag‐specific CD4^+^ T cells are required for activation of APCs in the presence of non‐immunogenic ALL Ags. Additionally, these results suggest that the central tolerance to leukemia‐associated antigens that is likely generated by the occurrence of the initiating genomic events in utero, limits the degree of protection achieved by an ALL‐targeted T cell response induced later in life.

APC licensing by CD4^+^ T cells requires recognition of the MHC II‐peptide complex by the T cell receptor. Thus, we tested expression of MHC II on cDCs from mice 20 days after challenge with ALL. Compared to leukemia‐naïve mice, MHC II was strongly downregulated in BALB/c mice injected with wt‐ALL (Figure 2E). MHC II expression on cDCs from responsive GFP/luc‐ALL challenged BALB/c mice was unchanged compared to naïve mice (Figure 2E). Antibody blockade of MHC II reduces IL‐12 production in the presence of leukemia‐responsive CD4^+^ T cells down to the level of naïve T cells (Figure 2F). This supports that direct DC‐CD4^+^ T cell interactions are essential to license DCs in the presence of non‐immunogenic leukemia Ags. Whether licensed DC are ultimately able to promote naïve T cell activation was evaluated by IFN‐y ELISPOT assay. Supernatants from conditions that produced the highest levels of IL‐12 were capable of activating naïve T cells (CD4^+^ and CD8^+^) to produce IFN‐y in the presence of wt ALL target cells, while supernatants from naïve BMDC cultures did not (Figure 2G). Further, T cells from wt ALL‐exposed mice had a significantly reduced response to co‐culture with wt ALL and supernatant from licensed APCs. This suggests licensed DCs are able to stimulate a broad anti‐ALL T cell response, but this capacity alone is insufficient to overcome T cell unresponiveness resulting from early expoure to non‐immunogenic leukemia Ags or over‐expression of IC.

Together our results reveal a CD4^+^ T cell‐mediated mechanism of professional APC licensing that broadens anti‐ALL T cell responses to include otherwise non‐immunogenic Ags. The immune response to xenoAgs presented by ALL blasts is characterized by downregulation of negative checkpoint regulators, which have been implicated in immune escape from single antigen‐targeted therapies. In our model, non‐immunogenic Ags are recognized when presented in combination with immunostimulatory Ags or in the presence of TLR agonists, which may have translational relevance to patients. However, inherent immune unresponsiveness to leukemia‐associated Ags, mediated through tolerance or exhaustion pathways, may be a hurdle to clinical translation. Approaches to induce durable anti‐ALL responses by promoting immunogenic cross‐presentation of otherwise non‐immunogenic Ag may be applicable in the post‐stem cell transplantation setting or after antigen‐directed therapy, such as CAR‐T or bi‐specific T cell engagers against CD19, CD22, or CD123. In summary, these findings provide insights for improving the efficacy of single Ag‐targeted therapies by inducing recognition of non‐immunogenic tumor‐specific epitopes.

## Author Contributions

**Luis Gil‐de‐Gómez:** conceptualization (supporting), data curation (lead), formal analysis (lead), investigation (lead), methodology (equal), project administration (equal), supervision (equal), validation (lead), visualization (equal), writing – original draft (equal), writing – review and editing (equal). **Joseph J. Mattei:** investigation (supporting), methodology (supporting), validation (supporting). **Jessica H. Lee:** investigation (supporting), methodology (supporting), validation (supporting). **Stephan A. Grupp:** conceptualization (supporting), resources (supporting), visualization (equal). **Gregor S.D. Reid:** conceptualization (equal), investigation (supporting), methodology (supporting), visualization (equal), writing – original draft (equal), writing – review and editing (equal). **Alix E. Seif:** conceptualization (equal), data curation (supporting), formal analysis (supporting), funding acquisition (lead), investigation (supporting), methodology (equal), project administration (equal), resources (lead), supervision (equal), visualization (equal), writing – original draft (equal), writing – review and editing (equal).

## Ethics Statement

Institutional Animal Care and Use Committee—protocol IAC 18‐000232 (Stephan A. Grupp). Institutional Biosafety Committee—protocol IBC 2009‐01‐008 (Stephan A. Grupp). No human samples were used in this work.

## Conflicts of Interest

The authors declare no conflicts of interest.

## Supporting information


Figure S1.

## Data Availability

The data that supports the findings of this study are available in the supporting information of this article.
