# Correction to “CD4+ T Cells Mediate Dendritic Cell Licensing to Promote Multi‐Antigen Anti‐Leukemic Immune Response”

**DOI:** 10.1002/cam4.71261

**Published:** 2025-09-17

Gil‐de‐Gómez, L., Mattei, J.J., Lee, J.H., Grupp, S.A., Reid, G.S.D. and Seif, A.E. (2025), CD4+ T Cells Mediate Dendritic Cell Licensing to Promote Multi‐Antigen Anti‐Leukemic Immune Response. Cancer Med, 14: e70508. https://doi.org/10.1002/cam4.70508

Affiliation number 2 should include “IDIVAL.” It should have read: “Department of Molecular Biology, University of Cantabria School of Medicine‐IDIVAL, Santander, Cantabria, Spain.”

We apologize for this error.

