# Supplementary material for: CD4 + T Cells Mediate Dendritic Cell Licensing to Promote Multi‐Antigen Anti‐Leukemic Immune Response
Source: Cancer Med. 2024 Dec 27;14(1):e70508. doi: 10.1002/cam4.70508 (PMC11671796; doi:10.1002/cam4.70508)
Supplement: Supplementary file 1 — Figure S1. [file CAM4-14-e70508-s001.docx]

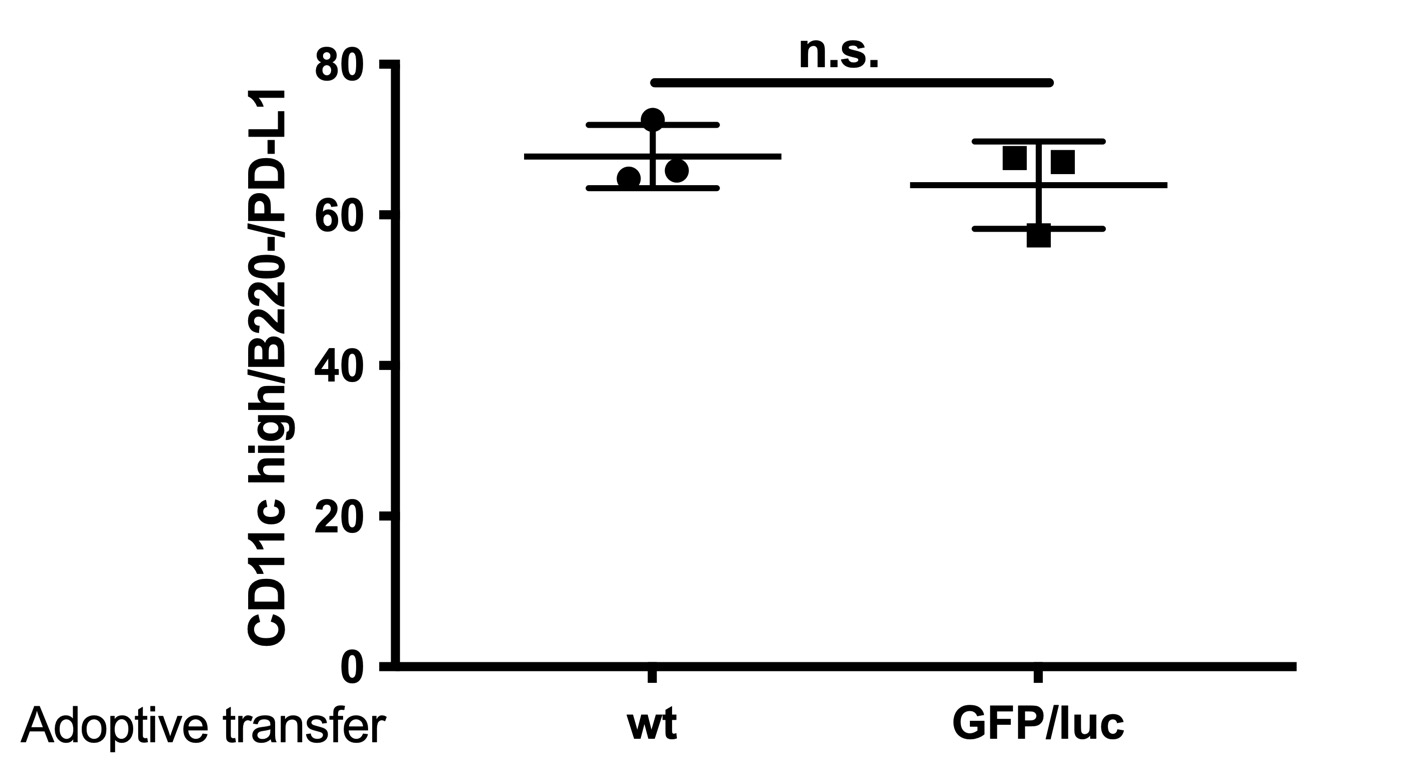


%

A

B

Supplementary figure 1. (A) Role of cDCs does not include ligation of PDL-1. No significant differences are found in PDL-1 expression on cDC harvested from ALL responsive and non-responsive mice at day 20 after tumor challenge. Unpaired t-test. (B) Non-immunogenic leukemia Ags do not induce robust APC responses in the absence of CD4^+^ T cells. DCs from GFP/luc ALL-responsive mice produce robust IL-12 in response to wt ALL cells either in presence or absence of CpG. One-way ANOVA. ***P <0.001

A


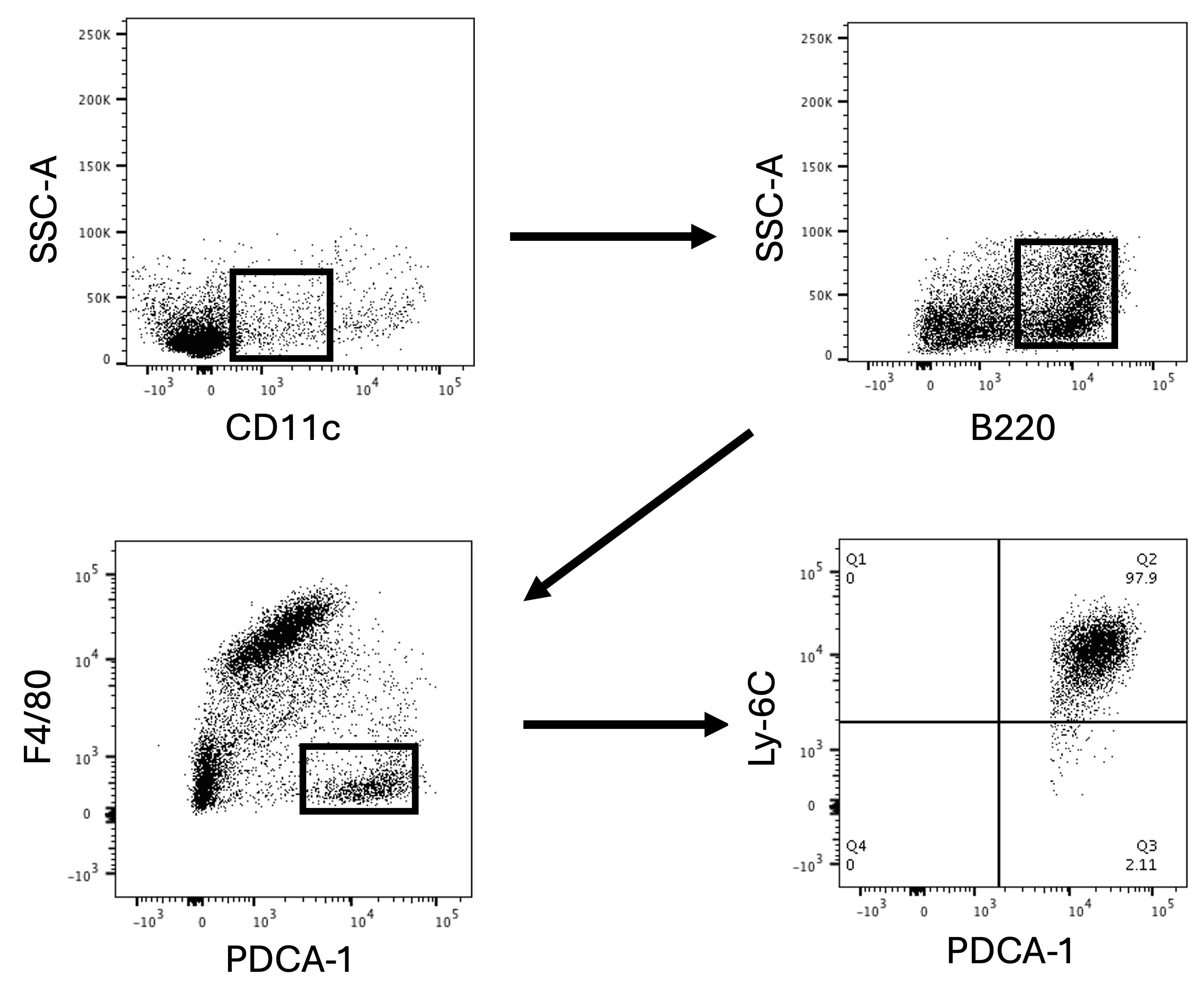


B


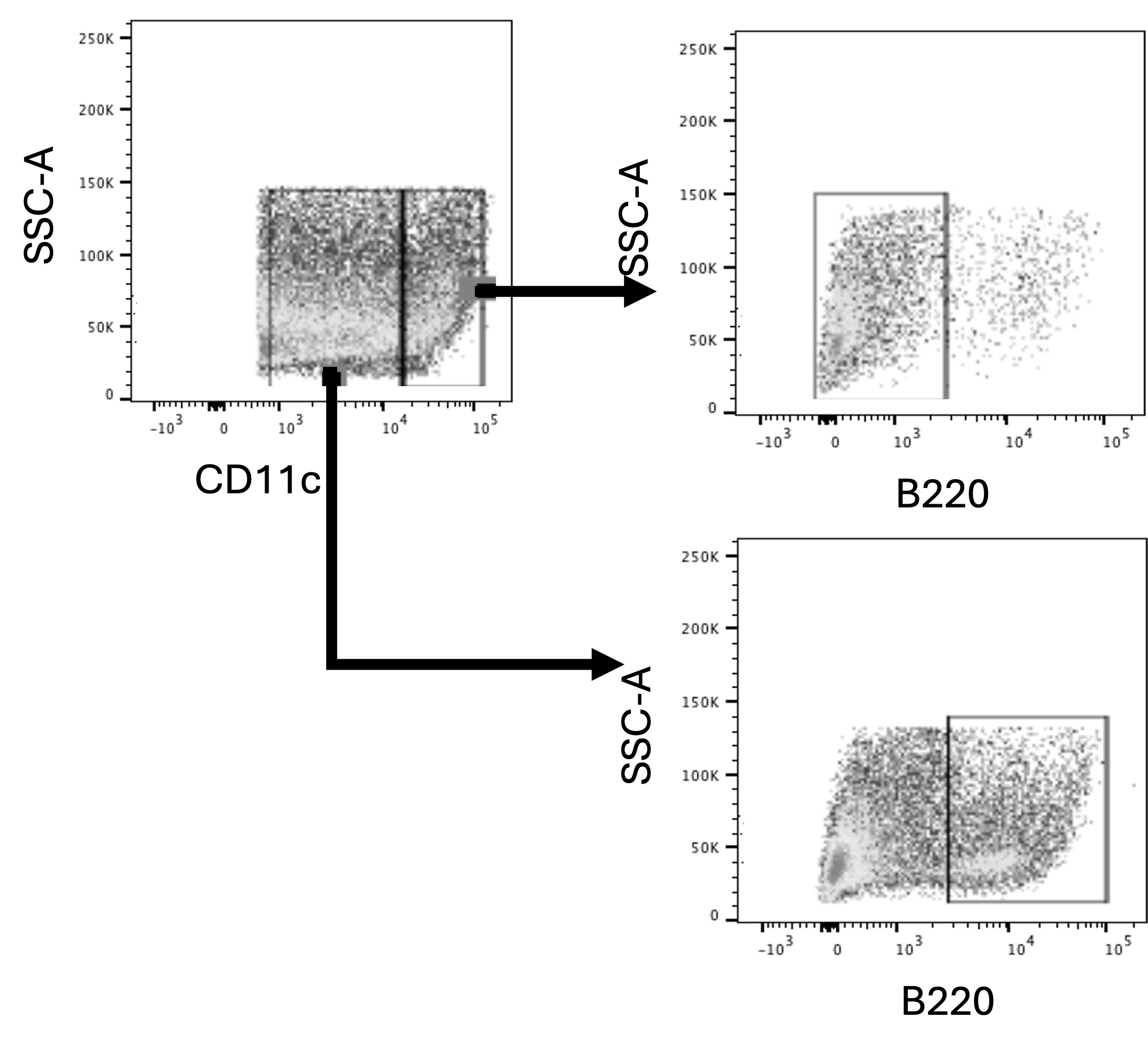


C


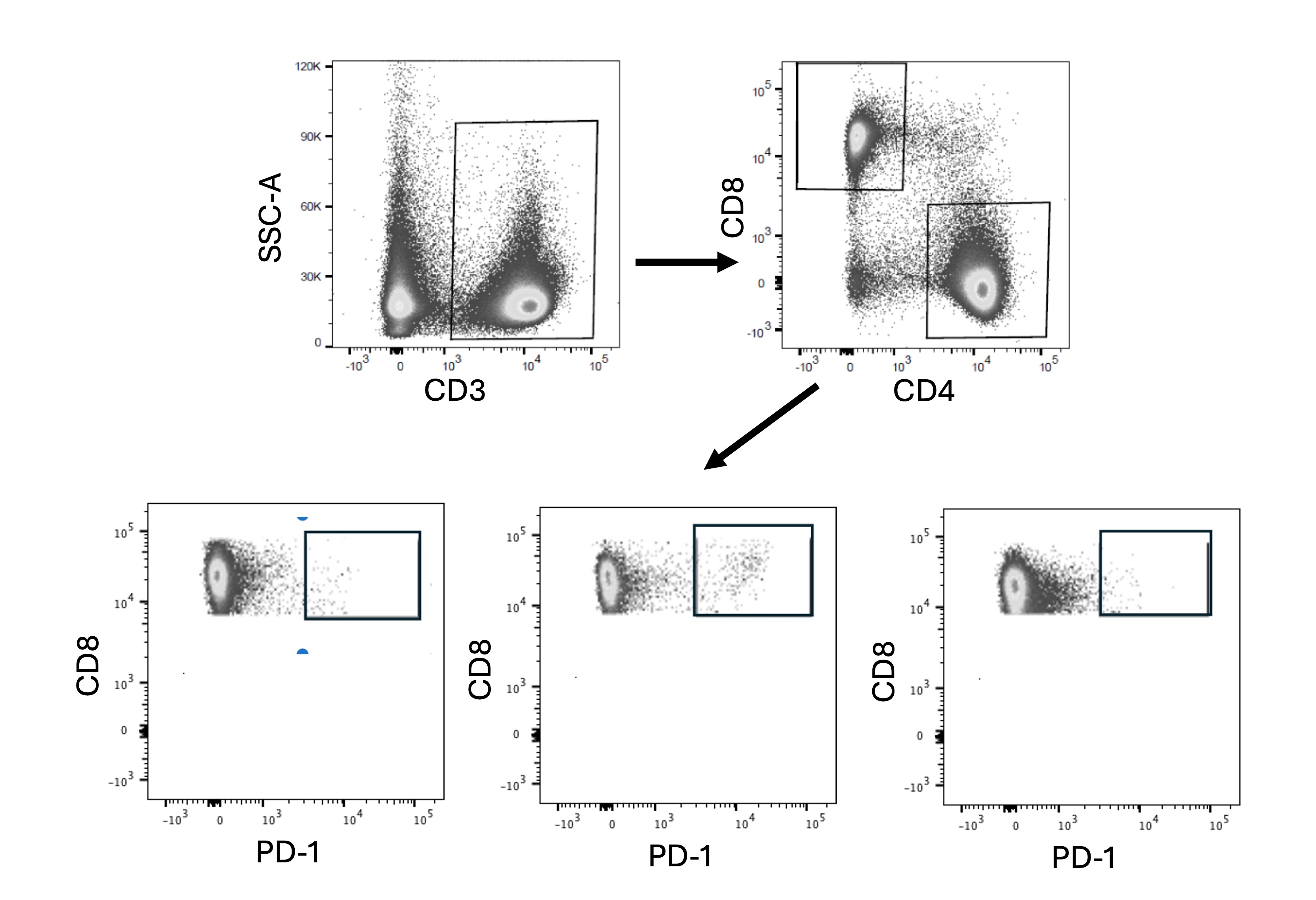


Supplementary Figure 2. (A) pDC are required for leukemia protective immune response. pDC are characterized by flow cytometry by CD11c int/B220^+^/F4/80^-^/PDCA-1^+^. To corroborate the identity of this population, staining of Ly-6C were performed in some of samples. 98% of CD11int/B220^+^/F4/80^-^/PDCA-1^+^ cells were LyC6C^+^ supporting the identification of this population. (B) Characterization and identification of cDC vs pDC. cDC are characterized by flow cytometry by CD11c hight/B220^-^. More than 90% of the events selected as CD11c high are also B220 negative. In contrast, pDC are characterized by CD11c int/B220^+^ which represent about 30% of total CD11c int population. (C) Characterization of CD4^+^ and CD8^+^ T cells populations and immune checkpoints expression. Expression of PD-1 was measured on CD3^+^/CD4^+^ T cells and CD3^+^/CD8^+^ T cells populations. PD-1 is overexpressed in CD8^+^ T cells from mice exposed to wt ALL at day 20.

**Sample sizes, replicates, and experimental repetitions**

| **Figure** | **Sample sizes or replicates** | **Number of experiments** |
| --- | --- | --- |
| 1A | n=15; Naïve BALB/c, n=5; Naïve BALB/c + unmodified ALL, n=5; Naïve BALB/c +GFP/luc ALL, n=5 | Representative study of 3 studies |
| 1B | n=12; Eμ-ret + = 3; Naïve BALB/c, n= 3; Eμ-ret + GFP/luc ALL, n= 3; Naïve BALB/c + GFP/luc ALL, n=3 | Combined results of 3 studies |
| 1C | n=6; Naïve BALB/c + wt ALL, n=3; Naïve BALB/c + GFP/luc ALL, n=3 | Representative study of 3 studies |
| 1D | n=6; Naïve BALB/c + wt ALL, n=3; Naïve BALB/c + GFP/luc ALL, n=3 | Representative study of 3 studies |
| 1E | n=6; Naïve BALB/c + wt ALL, n=3; Naïve BALB/c + GFP/luc ALL, n=3 | Representative study of 3 studies |
| 2A | Cell cultures plated in triplicate except BMDC + CD4^+^ T cells + CpG condition in duplicate. CD4^+^ T cells were pooled from n=5 Naïve BALB/c + GFP/luc ALL | Representative study of 2 studies |
| 2B | Cell cultures plated in duplicate. CD4^+^ T cells were pooled from BALB/c mice, n=3; and GFP/luc-transgenic mice, n=3 GFP/luc- | Representative study of 2 studies |
| 2C | Cell cultures plated in duplicate. | Representative study of 3 studies |
| 2D | Cell cultures plated in duplicate. CD4^+^ T cells were pooled from Naïve BALB/c + GFP/luc ALL, n=3; and GFP/luc-transgenic mice, n=3 | Representative study of 3 studies |
| 2E | n=9; Naïve BALB/c, n = 3; Naïve BALB/c + wt ALL, n=3; Naïve BALB/c + GFP/luc ALL, n=3 | Combined results of 2 studies |
| 2F | Cell cultures plated in triplicate except BMDC in absence of CD4^+^ T cells condition in duplicate. CD4^+^ T cells were pooled from n=5 Naïve BALB/c + GFP/luc ALL | Representative study of 2 studies |
| 2G | Cell cultures plated in triplicate for naïve T cells and in duplicate for both, T cells from n=3 pooled Naïve BALB/c + wt ALL and from n=3 pooled Naïve BALB/c + GFP/luc | Combined results of 2 studies |
